# Relationship between traditional maternal diet pattern and breastmilk composition of rural lactating women during the first month postpartum in Shigatse, Tibet

**DOI:** 10.1002/fsn3.2384

**Published:** 2021-07-03

**Authors:** Xiaomei Zhang, Minghui Zhang, Tie Lin, Junying Zhao, Zhang Luo, Juncai Hou, Baoguo Sun, Lijun Chen

**Affiliations:** ^1^ National Engineering Center of Dairy for Maternal and Child Health Beijing Sanyuan Foods Co. Ltd. Beijing China; ^2^ Beijing Engineering Research Center of Dairy Beijing Technical Innovation Center of Human Milk Research Beijing Sanyuan Foods Co. Ltd. Beijing China; ^3^ Beijing Advanced Innovation Center for Food Nutrition and Human Health Beijing Technology & Business University Beijing China; ^4^ Food Science College Tibet Agriculture & Animal Husbandry University Nyingchi, Tibet China; ^5^ Key Laboratory of Dairy Science, Ministry of Education College of Food Science Northeast Agricultural University Harbin China

**Keywords:** fatty acid, free amino acid, human breastmilk composition, macronutrients, Tibetan diet pattern

## Abstract

Maternal nutrition can influence the composition of human breastmilk by altering the components that are sensitive to maternal diet pattern. Traditional Tibetan maternal diet pattern among native rural lactating women possesses distinct characteristics due to its unique geographical environment and dietary habits. This study investigated maternal diet pattern and human breastmilk composition of Tibetan lactating women through different lactation stages during the first month postpartum in Shigatse. The results indicated that Tibetan maternal diet profile was apparently monotonous, mainly sufficient in Zanba, buttered tea, red meat, and fatty soup, yet insufficient in white meat, eggs, leafy vegetables, and fruits, leading to imperfect maternal nutritional intakes with high‐level carbohydrates and deficient proteins. Distinctions of maternal diet profiles in various degrees can be discovered upon different lactation stages, which brings multiple influences to the composition of human milk. There was significantly weak‐to‐medium correlation of protein contents between maternal diet intakes and human milk, while other macronutrients correlated insignificantly. Micronutrient constituents in human milk, involving functional unsaturated fatty acids and free essential amino acids, were also impacted by maternal diet intakes through different lactation stages. These results show that more systematic and profound research is requisite for the clarification and development of Tibetan maternal diet to offer more enhanced and individualized nutritional recommendations for Tibetan lactating women and infants.

## Graphical abstract

This study investigated maternal diet pattern and human breastmilk composition of Tibetan lactating women through different lactation stages during the first month postpartum in Shigatse. Distinctions of maternal diet profiles in various degrees can be discovered upon different lactation stages, which brings multiple influences to the composition of human milk. These results show that more systematic and profound research is requisite for the clarification and development of Tibetan maternal diet to offer more enhanced and individualized nutritional recommendations for Tibetan lactating women and infants.

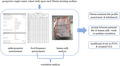

## 1 INTRODUCTION

Human breastmilk has always been the most idealistic natural nutritional source to meet the developmental needs of infants (Kramer & Kakuma, 2004). World Health Organization (WHO) has encouraged lactating women to breastfeed exclusively at the first 6 months to adequately ensure infants' multiple nutrients and bioactive components(Andreas et al., 2015; santé et al., 2003). The composition of human milk is a complex and dynamic system, which is affected by various internal and external elements. Among the influencing factors, maternal diet pattern has been stated to play an important role on the variation of breastmilk composition, especially on the essential components that are hard to be synthesized by human own metabolism, such as essential fatty acids and amino acids (Ding et al., 2010; Kim et al., 2017).

Macronutrients, that is, fats, proteins, and carbohydrates, constituent the primary composition of human milk, which are the most abundant nutritive portion and inferior to the proportion of water in human milk (Giuffrida et al., 2019). Thereinto, milk fat is the main source of energy for infants. Meanwhile, milk fat also provides functional polyunsaturated fatty acids (omega‐3 and omega‐6 PUFA) and lipid‐soluble vitamins (A, D, E, K), as well as influences the taste and mouthfeel quality of human milk (Koletzko et al., 2011). Moreover, the content and composition of milk fat are more variable than some other human milk components (such as carbohydrate), comparatively more sensitive to internal and external factors from mothers, infants, or lactation stages (Demmelmair & Koletzko, 2018). Maternal diet has been indicated to be correlated with the fat composition in human milk in previous publications, especially regarding the association of functional PUFA between human milk and maternal diet (Aumeistere et al., 2018; Jiang et al., 2016; Visentin et al., 2019; Yuhas et al., 2006). On the other hand, free amino acids (FAAs) were an important moiety of nonprotein nitrogen fraction in human milk, which promote the utmost utilization of dietary nitrogen and the regulation effect on infant early postnatal development due to the higher absorption efficiency of FAAs than protein‐derived amino acids (Agostoni et al., 2000; Zhang et al., 2013). In particular, certain specific amino acids from FAA in human milk can exert more impact on the initialization of relevant biological functions during infancy, involving protein synthesis, appetite control, and growth regulation (Agostoni et al., 2000; Elmastas et al., 2008). The influence of maternal diet on the level of nitrogen fraction in human milk has been discussed earlier than the 1970s with controversial viewpoints, especially on the association of protein contents between maternal diet and human milk (Boniglia et al., 2003; Wurtman & Fernstrom, 1979). Albeit such consequences, some nitrogen proportions in human milk (e.g., essential amino acids, whey‐to‐casein ratios, certain functional proteins) were considered to be correlated with maternal nutritional intakes, which could be more obvious among malnutrition women (Boniglia et al., 2003).

Tibet of China, noted as “the third pole of the world,” lies in the southwest of the Tibetan Plateau with an average altitude of over 4,500 m, which is the highest plateau of the world (Xie et al., 2014; Yang et al., 2016). Because of its peculiar geographical location, formidable climatic conditions, and unique traditional customs, the local maternal and child nutritive style possesses distinct characteristics in various aspects. However, the exploration and development of relevant maternal and child health research has remained limited, especially on the maternal breastfeeding and infant development, which should be due to the inconvenience of resource utilization and mutual communication by the extreme natural conditions. Shigatse, the second largest city of Tibet, locates between the middle part of the Himalayas and the middle part of the Gangdise‐Nyenchen Tanglha Mountains, within which stands Mount Everest (Yang et al., 2016). The meaning of “Shigatse” in Tibetan language is a great farm with the best soil, indicating its advantage of agricultural output (Yang et al., 2016). Meanwhile, Shigatse has a population of about 850,000, 89.9% of which is Tibetan and over 80% is farmers and herdsmen. Hence, the diet pattern of local lactating women is presumably capable to present the traditional Tibetan maternal dietary feature in nowadays.

Through the investigation of Tibetan maternal diet habit, the anthropometric measurement of local lactating women, and the analysis of human milk components during the first month postpartum were performed via prospective single‐center cohort study. The aim of this research was in an effort to explore the association between the Tibetan maternal diet pattern and the human milk composition, which hoped to be conducive to the reveal of maternal breastfeeding status and the development of local maternal and infant nutritional level at high‐altitude areas with traditional culture.

## 2 MATERIALS AND METHODS

### 2.1 Participant recruitment

The prospective single‐center cohort study was conducted from June 2018 to February 2019, which had acquired prior approval from the Ethics Committees of the Chinese Capital Institute of Pediatrics (SHERLL2014034) according to Helsinki declarations involving human subjects. At the beginning of the study, participants were recruited through random invitations at the local maternal and child clinics in Shigatse. After individual assessment and face‐to‐face interviews, 50 healthy (free of chronic/genetic/infectious diseases) expectant mothers, who were largely anticipated to deliver healthy term infants, were chosen from the local Tibetan applicants in various rural sections of Shigatse. Emphatically, the recruited mothers were eligible for the criteria of native residency in Shigatse, restriction on Tibetan diet tradition, and individual will and capacity in breastfeeding.

### 2.2 Questionnaire

With assistance from our trained and experienced researchers, basic information and dietary items were obtained from the recruited mothers during every sampling period on personal sociodemographic characteristics, physical status, dietary intakes, medical histories, and other necessities. Explicitly, maternal relevant data were recorded at the following time points: (a) necessary personal information acquired at postpartum periods among 0–5 days (colostrum stage), 7–15 days (transitional milk stage), and 26–30 days (mature milk stage) when breastmilk samples were collected; and (b) detailed dietary intakes recorded for 3 days before the collection of breastmilk samples. A 32‐diet‐item food frequency questionnaire (FFQ) with practicable validity and reproducibility was adopted for the diet investigation of local lactating women. The items of food products were divided into 12 groups according to previous relevant reports and local Tibetan diet habits in Shigatse (Cheng et al., 2009; Z. Wang et al., 2010). The feedback of FFQ was consisted of the frequency and consumption quantity of a certain food or beverage, and its existing form in certain relevant recipe. The evaluation of food intakes by FFQ was aimed to assess the level of nutrition intakes of local lactating women with adherence to traditional Tibetan diet pattern. Macronutrient intakes of carbohydrate, protein, fat, and energy were calculated according to the 2017 China Food Composition Tables (INFS, C. C., 2017), corresponding to the items of macronutrients in human milk analysis.

### 2.3 Breastmilk sample collection

After finishing 3 × 24 hr daily diet questionnaires, participants were asked to collect complete either‐side breastmilk by electric breast pump in the next forenoon between 09:00 a.m. and 11:00 a.m. in a sanitary and quiet environment. 60‐ml aliquot of the well‐shaken breastmilk was then divided equally into 10‐ml sterilized tubes. All the breastmilk samples were prestored at −20°C for no more than 7 days before being transported by ice box to −80°C refrigerators until analysis.

### 2.4 Analysis of human milk samples

#### 2.4.1 Macronutrient content analysis

The macronutrients of fat, crude protein, lactose, total solids, true protein, and energy in human milk samples were measured by human milk analyzer (MIRIS HMA, MIRIS AB, Uppsala, Sweden) with XMA‐SW software version 2.87. MIRIS HMA determined the macronutrient contents of human milk based on semi‐solid mid‐infrared transmission spectroscopy under different waveband conditions, which can be specifically as follows: 5.7 μm for functional carbonyl groups (fat content determination), 6.5 μm for amide groups (protein content determination), and 9.6 μm for hydroxyl groups (carbohydrate content determination). According to the recommended procedure from the manufacturer, human milk samples, which were stored at −80℃ before analysis, should be homogenized for 30 s by ultrasonic probe (MIRIS Sonicator, MIRIS AB) after being preheated to 40°C. Prior to analysis, preparation steps of self‐calibration check and autocleaning check were performed between every continuous 10th tests or without measurement within 5 min. Prepared human milk sample (3 ml) was injected into the flow cell and started to test immediately. Within 1 min, the test data would be shown on the system software.

#### 2.4.2 Fatty acid analysis

The composition of fatty acids in human milk was analyzed by gas chromatography‐mass spectrometry (GC‐MS) with the pretreatment of *n*‐hexane extraction and transesterification by methanol. Milk sample (250 μl) was mixed with 5 ml HCl–methanol (1:1, 0.5 mol/L) solution, 2 ml *n*‐hexane, and 2 ml methanol. The mixture was incubated at 80°C for 2 hr with mechanical stirring, which was cooled to room temperature by flowing water afterward. With the addition of 2 ml deionized water, the mixture was centrifuged at 2352 *g* for 5 min. Finally, 1 ml of liquid supernatant was extracted for GC‐MS analysis. Fatty acid methyl esters (FAMEs) were detected using GC‐MS (Thermo Trace 1300‐Thermo ISQ LT, Thermo Scientific) with an autosampler (Thermo Scientific TriPlus RSH). 1 μl of FAMEs was injected at 200°C into a HP‐88 capillary column (100 m × 0.250 mm × 0.20 μm) at a 10:1 split ratio. Nitrogen was used as carrier gas at the flow rate of 1 ml/min. The oven temperature program was set as follows: the initial time was programmed at 60°C and held for 5 min, then increased at 8°C/min to 160°C, next increased at 4°C/min to 200°C and held for 5 min, and finally increased at 3°C/min to 240°C and held for 5 min. The solvent delay was 10 min. The temperature of ion source and interface was 280°C and 240°C, respectively. The mass scan parameters included electron impact ionization voltage of 70 eV and mass range of 35–400 amu. The identification of FAMEs was achieved by comparison of their mass spectra with the corresponding authentic chemicals and the standard spectra of the SWGDRUG MS library with a match of 98%. Calibration and quantitation analysis were determined with the Xcalibur Data System using mixed external standard fatty acid solutions of spiked concentrations, which were 1, 2, 4, 8, 16, 24, and 32 mg/L.

#### 2.4.3 Free amino acid analysis

The composition of free amino acids in human milk was analyzed by ultraperformance liquid chromatography (UPLC) with photo‐diode array (PDA) detector using AccQ·tag Ultra C18 1.7 μm, 2.1 × 100 mm column, and AccQ‐Fluor Reagent Kit (all from Waters Corporation) for precolumn derivatization. Milk sample (300 μl) was mixed with 20 μl 10% sulfosalicylic acid in a 1.5‐ml tube, which was then centrifuged for 15 min at 9408 *g* and 4°C and filtered through 0.22‐μm ultrafiltration membrane. 10 μl filtrate was mixed with 70 μl borate buffer (0.4 M) and 20 μl AccQ‐Fluor in a UPLC tube, which was incubated at 55°C for 10 min and then cooled to ambient temperature for UPLC analysis. The same method was applied for the standard solutions (2.5 μmol/ml) of 17 amino acids, which was histidine, serine, arginine, etc. 1 μl of derivatized samples was injected into the UPLC system, which was detected by PDA detector at 260 nm. The solvent used for mobile stage was the manufacturer‐supplied AccQ‐Tag Ultra Eluent A and AccQ‐Tag Ultra Eluent B. The UPLC gradient program (A = AccQ‐Tag Ultra Eluent A, B = 10%AccQ‐Tag Ultra Eluent B, C = water, and D = AccQ‐Tag Ultra Eluent B) is listed in Table 1.

**TABLE 1:** The UPLC gradient program

Time (min)	Flow (ml/min)	A (%)	B (%)	C (%)	D (%)
0.00	0.7	10.0	0.0	90.0	0.0
0.29	0.7	9.9	0.0	90.1	0.0
5.49	0.7	9.0	80.0	11.0	0.0
7.10	0.7	8.0	15.6	57.9	18.5
7.30	0.7	8.0	15.6	57.9	18.5
7.69	0.7	7.8	0.0	70.9	21.3
7.99	0.7	4.0	0.0	36.3	59.7
8.59	0.7	4.0	0.0	36.3	59.7
8.68	0.7	10.0	0.0	90.0	0.0
10.20	0.7	10.0	0.0	90.0	0.0

### 2.5 Statistical analysis

All the experiments were performed in triplicate. The results from the analysis of macronutrient contents, fatty acid composition, and free amino acid composition were determined by the analysis of nonparametric variance (Kruskal–Wallis test) using SPSS (version 22.0; SPSS Inc.). The results were regarded significantly different at *p* < .05. The results of correlation analysis between maternal daily diet and human milk upon macronutrients were processed by the analysis of nonparametric bivariate correlation (Spearman's rho test) using SPSS. The results of correlation were considered as highly strong correlation when *p* < .05, .8 < |*r*| < 1; strong correlation when *p* < .05, .6 < |*r*| < .8; medium correlation when *p* < .05, .4 < |*r*| < .6; weak correlation when *p* < .05, .2 < |*r*| < .4; and uncorrelation when *p* < .05, 0 < |*r*| < .2 or *p* > .05. Principal component analysis (PCA) was used to determine the differences of maternal diet profile through three lactation stages by Unscrambler X 10.4 (CAMO Inc.). Cluster heatmap analysis was performed to show the classification of free essential amino acids (i.e., *His*, *Leu*, *Lys*, *Phe*, *Val*, *Thr*, *Met*, *Ile*, and *Tau*) and main functional unsaturated fatty acids (i.e., linoleic acid, α‐linolenic acid, arachidonic acid, eicosapentaenoic acid, docosahexaenoic acid) by OriginPro 2018C attached to an app of Heat Map with Dendrogram (OriginLab Corporation).

## 3 RESULTS AND DISCUSSION

### 3.1 Maternal and infant characteristics in this cohort study

As shown in Table 2, 45 (45/50) expectant mothers managed to complete the thorough participation of this cohort study with enough supply of effective data and breastmilk samples. Specifically, 44 expectant mothers had healthy term infants and 1 had preterm infants, which were consisted of 21 baby boys and 24 baby girls. Meanwhile, other 5 (5/50) mothers failed to finish the participation for the reasons of miscarriage, incapable to supply complete breastmilk and data or other individual matters. The participants had a mean age of 27.21 years (*SD* = 4.48), which was fell into the range of prime ages for childbearing (Thalberg, 2011). The mean prepregnancy BMI was 21.66 kg/m^2^ (*SD* = 2.20). 35 participants had managed to maintain fit and fine for pregnancy. According to our detailed records, the main symptoms of other 10 participants during pregnancy were mild anemia and muscle cramp, which might be due to some malnutrition causes, such as iron or calcium deficiency. Before or during the pregnancy period, 37 participants used nutritive supplements, most of which were folic acid, iron supplement, or blood‐supplementing formula. Prior to our study criteria, the occupations of 46 recruited pregnant women were local Tibetan farmers and herdsmen, for a better assurance in reflecting traditional Tibetan diet pattern. 44 infants were delivered by natural vaginal mode, and 1 was through cesarean section. The mean birthweight and birth length of infants were 2,984.4 g (*SD* = 418.6) and 48.4 cm (*SD* = 4.0), respectively.

**TABLE 2:** Description of maternal and infant population

Characteristics of mothers	
Age of mothers (years): mean ± *SD*	27.21 ± 4.48
Prepregnancy BMI (kg/m^2^): mean ± *SD*	21.66 ± 2.20
Gestational age at birth (weeks): *n*	
≥37 weeks	44
<37 weeks	1
Ill categories: *n*	
Illness during pregnancy	10
No illness during pregnancy	35
Occupation: *n*	
Farmer & herdsman	46
Other occupations	2
Unemployment	2
Nutritional supplement for pregnancy: *n*	
Yes	37
No	13

Characteristics of infants	
sex: *n*	
Male	21
Female	24
Mode of delivery: *n*	
Vaginal	44
Cesarean section	1
Birthweight (g): mean ± *SD*	2,984.4 ± 418.6
Birth length (cm): mean ± *SD*	48.4 ± 4.0

### 3.2 Characteristics of Tibetan diet pattern among lactating women in Shigatse

The description of diet pattern among Tibetan lactating women in Shigatse is revealed in Table 3. Summarily, the selection of certain food types has basically possessed a shared commonness by Tibetan diet tradition, with some varied characteristics by individual maternal preference during the lactation period. Specifically speaking in food categories, Zanba (a traditional Tibetan food, which is cooked by roasting highland barley flour, usually mixed with buttered tea or red meat broth (Qu et al., 2015)) was the dominating cereal type for staple food, consumed by over 98% participants through all three lactation stages. Other cereal choices included noodle, rice, and deep‐fried dough stick. Particularly, Zanba seemed to be an exclusive choice of cereal among lactating women in colostrum stage, while the profile of cereal category would exhibit more diversity in the following transitional milk and mature milk stages. Moreover, Zanba was also used as the first type of cereal introduced to local Tibetan infants in about 1 month after birth according to previous publications (Dang et al., 2005). Such priority to Zanba in local maternal and infant diet pattern should be owed to the long‐held traditional diet culture among native Tibetans at all ages, especially in the remote rural areas (Wang et al., 2010). High‐fat drinks of broth (soup of beef, mutton, or bone) and Tibetan buttered tea were another traditional dietary category commonly included in the daily diet of the participants. Using a native way from the unique Tibetan tradition, the cooking of Zanba with broth or Tibetan buttered tea is a typical main course in their daily life. As revealed in this study, the intake frequency of the combination of Zanba and broth or Tibetan buttered tea was approximately at least once per day for about 80%–90% participants, many of whom even consumed more than once in their everyday diet. Other traditional Tibetan teas, such as light black tea (pure black tea) and sweet tea (Tibetan milk tea), were the most common beverages for Tibetan lactating women in this cohort study. Unexpectedly somewhat, highland barley liquor was involved in the diet of some participants during their lactating period, which was quite surprising for the reason that the alcohol ingested by mothers might transfer and bring adverse healthy implication to infants. However, some researchers have considered that low‐level alcohol consumption during breastfeeding might not presumably linked with the potential adverse outcomes if manageable breastfeeding strategies can be adopted (Wilson et al., 2017). Red meat, generally including beef and mutton, were the mainly consumed meat among the participants, which were usually cooked with Zanba and buttered tea or other traditional Tibetan staple foods, while white meat, such as poultry meat and fish, was rarely involved in their daily diet. Such inclination to red meat rather than white meat might be due to the high‐altitude environment and traditional Tibetan diet culture. Moreover, eggs were not a commonly involved food category in the participants' recipe, possibly on the account of less adoption upon poultry farming than livestock farming in such areas (Shang et al., 2014). It is unsurprisingly that pure milk was not taken as a drink for the lactating women, since milk was conventionally used as the ingredient for traditional Tibetan drinks and beverages, such as sweet tea. Vegetables and fruits were not a daily acquisition for every lactating mother, especially in the colostrum stage. Additionally, the ordinary vegetables were root and tuber vegetables, such as turnips, carrots, tomatoes, and potatoes, while leafy vegetables and fruits were taken less frequently, which were generally spinach, Chinese cabbage, pakchoi, and apple and banana according to our detailed information.

**TABLE 3:** Diet frequency of food products among participants during lactation

Food products	Colostrum stage			Transitional milk stage			Mature milk stage		
	Every day^a^	Sometimes^a^	Never^a^	Every day	Sometimes	Never	Every day	Sometimes	Never
Zanba	94%	6%	0%	88%	10%	2%	92%	6%	2%
Other cereals	16%	40%	44%	72%	26%	2%	84%	10%	6%
Fatty soup/buttered tea	94%	6%	0%	82%	18%	0%	82%	16%	2%
Black tea/sweet tea	52%	38%	10%	20%	38%	42%	28%	42%	30%
Red meat	82%	16%	2%	70%	26%	4%	74%	20%	6%
White meat	4%	12%	84%	2%	14%	84%	0%	16%	84%
Highland barley liquor	4%	22%	74%	14%	26%	60%	8%	36%	56%
Eggs	4%	36%	60%	18%	52%	30%	20%	50%	30%
Milk	4%	22%	74%	2%	48%	50%	4%	24%	72%
Leafy vegetables	6%	24%	70%	6%	46%	48%	6%	60%	34%
Other vegetables	4%	30%	66%	18%	54%	28%	24%	54%	22%
Fruits	6%	34%	60%	6%	44%	50%	2%	48%	52%

^a^ The expression of “every day,” “sometimes,” or “never” means a certain food was taken every day, no less than once and no more than every day, or never taken by participants during the 72‐hr sampling time.

Consequently, the lactating women in the rural areas of Shigatse were customarily prone to a traditional Tibetan dietary pattern, invariably including Zanba, Tibetan buttered tea and sweet tea, red meat, fatty soup, and root and tuber vegetables with the insufficiency of leafy vegetables, fruits, and white meat, which was in accordance with the findings of previous relevant reports (Ge et al., 1997; Wang et al., 2010). Referring to the advice from Chinese dietary guidelines, lactating mothers are recommended maintaining an assorted, balanced and moderate diet profiles, which consist of cereals (whole grains and legumes included), leafy and colored vegetables, fruits, various meats (white and red meats and seafood included), dairy products, nuts, etc. (Wang et al., 2016). Moreover, the diet patterns of lactating mothers in low‐altitude regions of China, especially urban areas, are more diversified and individualized than in Shigatse, yet with certain deficiencies in some nutrients (Ding et al., 2020; He et al., 2015; Huang & Hu, 2020; Wang et al., 2020).

### 3.3 Contents of macronutrients in human milk from Shigatse

The macronutrient contents of fat, crude protein, lactose, total solids, true protein, and energy in human milk are shown in Table 4, which were measured by MIRIS HMA human milk analyzer. The calibration of HMA is based on established standard methods, which are universally used with the recommendations of ISO (International Organization for Standardization) and IDF (International Dairy Federation), such as the Röse–Gottlieb method for fat content determination, and the Kjeldahl method for crude protein and true protein (crude protein minus nonprotein nitrogen) (Giuffrida et al., 2019). The contents of crude protein, lactose, and true protein in human milk were significantly different (*p* < .05) between the three lactation stages of colostrum, transitional milk, and mature milk. Similar to previous relevant reports, protein and true protein content of human milk decreased with the progress of lactation, especially between the stages of colostrum and transitional milk (Bauer & Gerss, 2011; Larnkjaer et al., 2006). Meanwhile, the contents of fat, total solids, and energy in the Shigatse human milk samples were insignificantly different (*p* > .05) during the lactation period. The level of lactose in human milk samples was nonmonotonously increasing/decreasing during lactation.

**TABLE 4:** Macronutrient contents in human milk

	Fat (g/100 ml)	Crude protein (g/100 ml)	Lactose (g/100 ml)	Total solids (g/100 ml)	True protein (g/100 ml)	Energy (kcal/100 ml)
Colostrum	1.9 ± 1.0	1.6 ± 0.4a^a^	5.0 ± 0.7b	8.7 ± 1.5	1.3 ± 0.4a	44.7 ± 11.3
Transitional milk	2.2 ± 1.0	1.4 ± 0.5b	5.4 ± 0.6a	9.3 ± 1.3	1.2 ± 0.4ab	48.6 ± 10.2
Mature milk	2.3 ± 1.4	1.4 ± 0.7b	5.2 ± 0.8ab	9.0 ± 2.2	1.1 ± 0.6b	47.8 ± 15.9
*p* value	.210	.003	.043	.114	.004	.165

Note

Values are means ± *SD*.

^a^ Different lowercase letters in a column represent statistical differences among samples, *p* < .05.

### 3.4 Composition of fatty acids in human milk from Shigatse

The fatty acid composition of human milk from Shigatse is displayed in Table 5. 36 fatty acids were detected in the human milk samples, including 17 saturated fatty acids (SFAs), 8 mono‐unsaturated fatty acids (MUFAs), and 11 polyunsaturated fatty acids (PUFAs). Significant differences (*p* < .05) were observed among the contents of varied fatty acid categories, such as individual fatty acids (single SFA, MFA, and PUFA) and certain fatty acid ratio (ARA: DHA). Palmitic acid (C16:0) was the most abundant SFA and second most abundant fatty acids in all three lactation stages. The content of oleic acid (C18:1 *n*−9c) was of the highest level among all MUFAs and even all fatty acids in human milk of any lactation stage. The content of LA (linoleic acid, C18:2 *n*−6c) was the most abundant PUFA in human milk and increased significantly (*p* < .05). Through the three lactation stages, various functional unsaturated fatty acids, including ALA (α‐linolenic acid, C18:3 *n*−3), EPA (eicosapentaenoic acid, C20:5 *n*−3), and LA (linoleic acid, C18:2 *n*−6c) were increased significantly in content (*p* < .05), while DHA (docosahexaenoic acid, C22:6 *n*−3), inversely, was decreased significantly (*p* < .05) in content simultaneously. Particularly, DHA has been argued to be sensitive to dietary level, which is likely to perform obvious variation trends in content when maternal diet profiles alter. In this study, DHA contents in the human milk samples from Shigatse were lower than the samples from areas with different diet patterns, especially among the coastal regions with sufficient seafood, such as South Korea, Hangzhou of China, Philippines, and Japan (Jiang et al., 2016; Kim et al., 2017; Yuhas et al., 2006). Among the SFA category, butyric acid (C4:0), caproic acid (C6:0), caprylic acid (C8:0), capric acid (C10:0), lauric acid (C12:0), arachidic acid (C20:0), and lignoceric acid (C22:0) were found to have significant differences in content among the three lactation stages, especially between colostrum and transitional milk. Moreover, for the sums of certain fatty acid categories, including the sum of *n*−3 fatty acids, *n*−6 fatty acids, SFA, MUFA, PUFA, and TFA, no significant difference was observed through varying lactation stages. The above analytical results were consistent with the relevant findings of previous publications (Jiang et al., 2016; Thakkar et al., 2019). Additionally, the fatty acid profile of human milk in Shigatse was comparatively deficient than in Chinese low‐altitude regions, especially the urban or coastal areas, which might be partly due to different dietary preferences. It has been reported that lactating mothers in Chinese low‐altitude coastal areas possessed higher intake of long‐chain PUFA, including DHA and EPA, than the mothers in the inland areas, which was assumed to be influenced by different dietary habits (Jiang et al., 2016; Peng et al., 2009). Meanwhile, the intakes of certain FAs, such ARA, might be insignificantly impacted by the differences between diet patterns in China (Peng et al., 2009).

**TABLE 5:** Fatty acid composition in human milk

Fatty acids (mg/L)	Colostrum	Transitional milk	Mature milk	*p* value
C4:0 (butyric acid)	0c^a^	0.24 ± 1.21b	0.11 ± 0.13a	.000
C6:0 (caproic acid)	0.23 ± 0.17b	0.68 ± 1.25a	0.61 ± 0.41a	.000
C8:0 (caprylic acid)	1.52 ± 1.58b	3.58 ± 2.08a	3.45 ± 2.25a	.000
C10:0 (capric acid)	12.84 ± 13.34b	22.81 ± 15.97a	20.02 ± 13.66a	.000
C11:0 (undecylic acid)	0.22 ± 0.18	0.22 ± 0.18	0.26 ± 0.19	.423
C12:0 (lauric acid)	44.34 ± 38.04b	74.52 ± 47.84a	77.61 ± 52.04a	.000
C13:0 (tridecylic acid)	0.72 ± 0.45	0.82 ± 0.33	0.69 ± 0.41	.067
C14:0 (myristic acid)	71.33 ± 42.82	87.52 ± 48.2	88.06 ± 54.87	.137
C15:0 (pentadecylic acid)	8.62 ± 5.44	9.85 ± 4.84	8.55 ± 5.9	.154
C16:0 (palmitic acid)	325.79 ± 153.85	364.52 ± 181.85	356.65 ± 180.39	.579
C17:0 (heptadecylic acid)	11.14 ± 5.63	13.2 ± 6.43	10.7 ± 6.39	.130
C18:0 (stearic acid)	141.92 ± 72.17	150.81 ± 63.45	131.35 ± 64.29	.351
C20:0 (arachidic acid)	4.02 ± 3.01b	5.61 ± 2.39a	4.6 ± 2.28b	.022
C21:0 (heneicosanoic acid)	0.54 ± 0.13	0.61 ± 0.18	0.55 ± 0.16	.063
C22:0 (docosanoic acid)	1.38 ± 1.27a	0.32 ± 0.01b	0.32 ± 0.01b	.000
C23:0 (tricosanoic acid)	0.49 ± 0.01	0.49 ± 0.01	0.49 ± 0.01	.844
C24:0 (lignoceric acid)	0.34 ± 0.01b	0.35 ± 0.02a	0.34 ± 0.01ab	.025
C14:1 (tetradecenoic acid)	5.17 ± 3.35b	7.73 ± 3.56a	6.95 ± 5.23ab	.001
C16:1 (gaidic acid)	29.86 ± 21.48b	36.73 ± 24.01b	53.7 ± 33.91a	.000
C17:1 (heptadecenoic acid)	5.38 ± 3.23	6.54 ± 3.18	7.05 ± 4.3	.080
C18:1 *n*−9t (elaidic acid)	19.84 ± 11.03	20.05 ± 9.43	21.36 ± 11.02	.698
C18:1 *n*−9c (oleic acid)	396.88 ± 202.25	452.99 ± 223.03	468.11 ± 221.07	.253
C20:1 (eicosenoic acid)	19.79 ± 11.93	14.62 ± 9.21	17.77 ± 11.4	.054
C22:1 *n*−9 (erucic acid)	13.03 ± 16.73	12.68 ± 12.1	18.46 ± 18.09	.070
C24:1 (nervonic acid)	0.21 ± 0.11b	0.25 ± 0.1a	0.16 ± 0.03b	.000
C18:2 *n*−6t (linoelaidic acid)	2.41 ± 1.26	2.74 ± 1.24	2.52 ± 1.35	.317
C18:2 *n*−6c (LA)	108.74 ± 85b	125.5 ± 96.12ab	150.01 ± 107.08a	.038
C18:3 *n*−6 (γ‐linolenic acid)	1.28 ± 1.4b	2.78 ± 2.33a	3.09 ± 2.27a	.000
C18:3 *n*−3 (ALA)	15.52 ± 12.67b	16.96 ± 13.07b	21.31 ± 14.3a	.004
C20:2 (eicosadienoic acid)	7.69 ± 5.46	5.82 ± 4.14	5.94 ± 3.86	.214
C20:3 *n*−6 (dihomo‐γ‐linolenic acid)	5.74 ± 3.23	5.53 ± 2.98	4.72 ± 2.32	.354
C20:3 *n*−3 (homo‐α‐linolenic acid)	2.67 ± 2.79	1.43 ± 0.77	1.49 ± 0.73	.063
C20:4 *n*−6 (ARA)	9.73 ± 5.48	10.56 ± 5.82	9.16 ± 4.41	.660
C22:2 (docosadienoic acid)	1.03 ± 0.58a	0.72 ± 0.8b	0.33 ± 0.05b	.000
C20:5 *n*−3 (EPA)	2.45 ± 1.55b	2.49 ± 1.6b	3.61 ± 1.76a	.002
C22:6 *n*−3 (DHA)	5.31 ± 3.26a	4.9 ± 2.82ab	3.71 ± 1.94b	.046
ARA: DHA	1.91 ± 0.4c	2.24 ± 0.54b	2.55 ± 0.49a	.000
*n*−3 fatty acids	25.95 ± 17.66	25.79 ± 15.08	30.13 ± 17.28	.135
*n*−6 fatty acids	127.89 ± 92.89	147.11 ± 104.78	169.5 ± 114.54	.072
SFA	625.46 ± 307.7	736.16 ± 325.83	704.37 ± 350.65	.196
MUFA	490.16 ± 250.66	551.58 ± 266.09	593.55 ± 275.09	.174
PUFA	162.56 ± 112.43	179.44 ± 123.43	205.89 ± 133.26	.103
TFA	1,278.18 ± 640.34	1,467.17 ± 683.37	1,503.81 ± 712.27	.212

Note

Values are means ± *SD*.

Abbreviations: ALA, α‐ linolenic acid; ARA, arachidonic acid; DHA, docosahexaenoic acid; EPA, eicosapentaenoic acid; LA, linoleic acid; MUFA, mono‐unsaturated fatty acid; PUFA, polyunsaturated fatty acid; SFA, saturated fatty acid; TFA, total fatty acid.

^a^ Different lowercase letters in a row represent statistical differences among samples, *p* < .05.

### 3.5 Composition of free amino acids in human milk from Shigatse

As described in Table 6, 18 FAAs were detected in the human milk samples from Shigatse. *Glu* was the most abundant FAA among all FAAs during the lactation period. *Tau* was of the highest level among all essential FAAs and second highest among all FAAs in human milk samples. Specifically, it is suggested that free *Glu* in human milk has a down‐regulating effect on the appetite and growth of infants, and it can be served as a satiety signal corresponding to the receptors in the oral cavity and gastrointestinal tract during feeding, which might be beneficial to the infant obesity inhibition, and long‐term fitness (Larnkjær et al., 2016). The contents of individual FAAs found to have significant differences (*p* < .05) in varying degrees among the three lactation stages. In particular, the contents of *His*, *Tau*, *Ser*, *Arg*, *Glu*, and *Thr* were significantly different (*p* < .05) between colostrum stage and the other two lactation stages, while the content of *Ala* in colostrum was significantly different with mature milk, but indifferent (*p* > .05) with transitional milk. Nine essential FAAs, *His*, *Tau*, *Thr*, *Lys*, *Met*, *Val*, *Ile*, *Leu*, and *Phe*, were detected in human milk through all three lactation stages. The whole content of essential FAAs was decreased significantly (*p* < .05) during colostrum and transitional milk stages, and remained insignificantly variable (*p* > .05) in mature milk stage. The content of total free amino acids was similar with the variation trend of essential FAAs through the lactation period, which was consistent with the results from other researches (Garcia‐Rodenas et al., 2016; Zhang et al., 2013).

**TABLE 6:** Free amino acid composition in human milk

AA (mg/L)	Colostrum	Transitional milk	Mature milk	*p* value
*His*	20.88 ± 14.04a^a^	12.46 ± 8.85b	12.00 ± 8.72b	.000
*Tau*	259.09 ± 102.66a	206.47 ± 98.03b	213.83 ± 106.5b	.032
*Ser*	42.21 ± 21.29a	36.15 ± 31.71b	32.77 ± 30.67b	.011
*Arg*	20.48 ± 31.12	7.67 ± 13.06	6.75 ± 8.51	.255
*Gly*	13.9 ± 8.31	11.62 ± 5.66	11.37 ± 5.16	.379
*Asp*	16.4 ± 14.31a	10.09 ± 7.6b	8.53 ± 6.56b	.004
*Glu*	513.31 ± 230.57a	353.16 ± 194.9b	305.84 ± 175.74b	.000
*Thr*	22.82 ± 10.87a	17.08 ± 7.08b	14.44 ± 7.15b	.000
*Ala*	24.05 ± 10.79a	19.81 ± 10.2ab	16.26 ± 8.23b	.002
*Pro*	17.14 ± 27.15	9.71 ± 11.09	11.63 ± 14.73	.973
*Cys*	4.16 ± 2.06	3.38 ± 2	3.4 ± 2.36	.080
*Lys*	12.52 ± 20.74	11.64 ± 17.71	10.61 ± 13.93	.483
*Tyr*	10.52 ± 18.67	7.88 ± 9.79	6.89 ± 8.69	.834
*Met*	8.47 ± 18.13	5.14 ± 10.18	3.35 ± 5.15	.554
*Val*	16.33 ± 10.58	13.81 ± 9.09	13.64 ± 11.19	.072
*Ile*	7.53 ± 13.74	4.51 ± 5.14	4.55 ± 5.78	.631
*Leu*	13.99 ± 14.23	13.79 ± 22.38	12.65 ± 17.64	.132
*Phe*	12.22 ± 22.01	9.52 ± 14.24	7.72 ± 7.97	.644
EAA	373.85 ± 138.88a	294.43 ± 125.6b	292.8 ± 133.81b	.003
TAA	1,036.03 ± 317.93a	753.9 ± 257.33b	696.24 ± 244.37b	.000

Note

Values are means ± *SD*.

Abbreviations: EAAs, essential amino acids, including *His*, *Leu*, *Lys*, *Phe*, *Val*, *Thr*, *Met*, *Ile*, and *Tau*; TAA, total amino acid.

^a^ Different lowercase letters in a row represent statistical differences among samples, *p* < .05.

### 3.6 Classification of maternal diet profiles through different lactation stages during the first month postpartum

**FIGURE 1:**
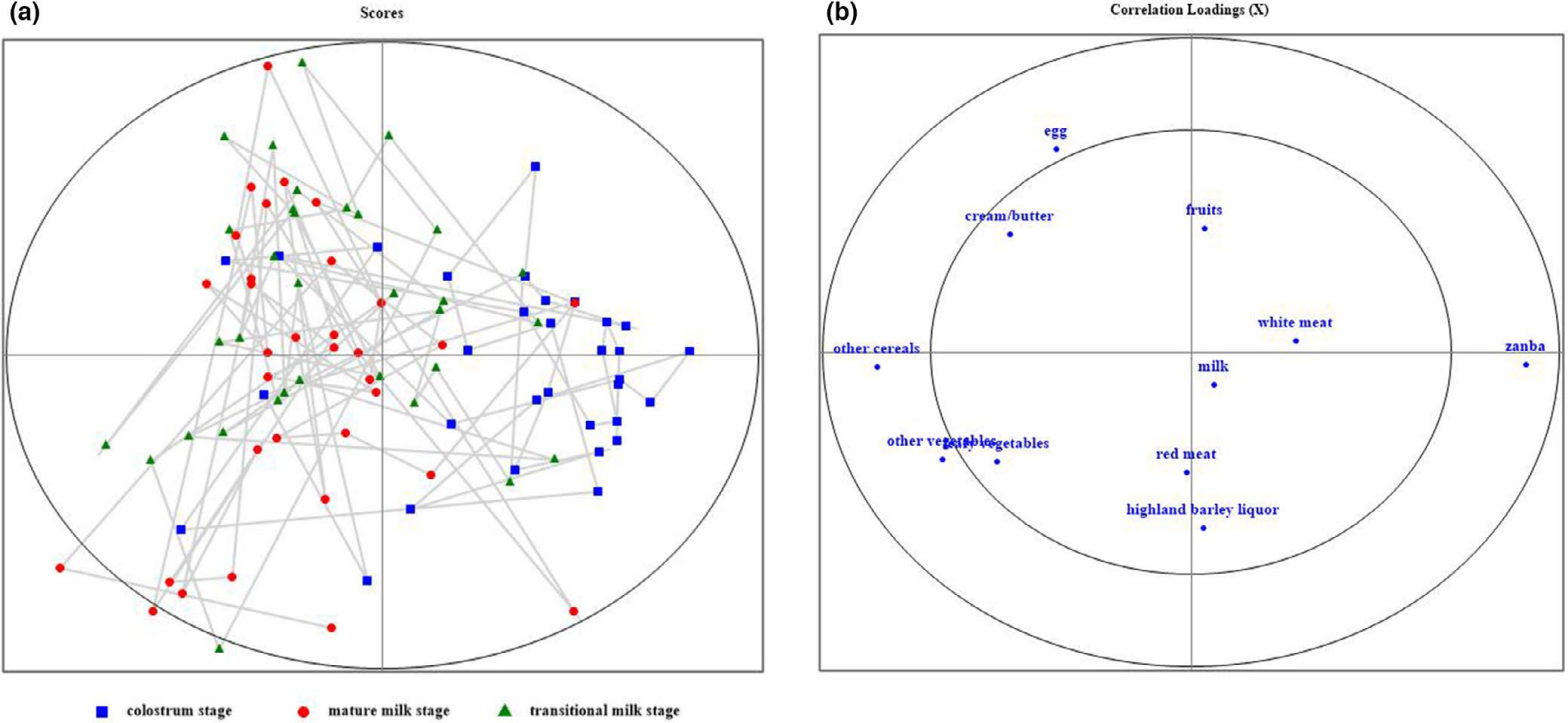
PCA and correlation analysis of stage differences in the food profile upon traditional Tibetan maternal diet pattern. (a) PCA score plot of food profiles in different lactation stages. (b) Correlation loadings of food products for PCA classification

Principal component analysis (PCA) was adopted to display the classification of maternal diet profiles through three lactation stages. From Figure 1a, the distribution of maternal diet profile in colostrum stage was obviously more centralized than that in transitional milk stage and mature milk stage, and the distributions of maternal diet profiles in transitional milk stage and mature milk stage were quite scattered within either category and some overlapped between the both categories. It might imply a more particular maternal diet profile in colostrum stage and some resemblance in diet profiles between transitional milk stage and mature milk stage. The contribution of certain food products to the classification of maternal diet profiles is shown in Figure 1b. The inner ellipse and outer ellipse revealed 50% and 100% of the explained variances, respectively. The food products of Zanba, other cereals (rather than Zanba), other vegetables (rather than leafy vegetables), and egg performed more correlative significance in the PCA determination of the lactation stage differences upon maternal diet profiles.

### 3.7 Correlation analysis of macronutrients between maternal diet profiles and human milk during different lactation stages

As shown in Table 7, the intake level of protein was lower than the recommended nutrient intake (RNI) of protein for Chinese lactating mothers; meanwhile, the intake level of carbohydrate was higher than the RNI of carbohydrate for Chinese lactating mothers. Besides, all the macronutrient intakes were fallen in the ranges of previous reports regarding the nutrient intakes of mothers from the relevant rural areas in western China (Cheng et al., 2009; Liu et al., 2015; Wang et al., 2010). Moreover, the intakes of energy and fat were through similar variation trends during the three lactation stages: initially held at significantly low (*p* < .05) levels in the colostrum stage, then increased significantly (*p* < .05) in the transitional milk stage and decreased significantly (*p* < .05) in the following mature milk stage. Furthermore, the intakes of protein were at low levels with insignificantly varying degrees (*p* > .05) through the lactation stages, while the intakes of carbohydrate increased significantly (*p* < .05) from colostrum stage to transitional milk stage and kept at a relatively steady level (*p* > .05) afterward. Correlated with the diet frequency of food products among participants (Table 6) and their living style, the above results of daily macronutrient intakes can be interpreted as follows: (a) dietary patterns—according to the Tibetan dietary habit, the main type of food products was the staple food, basically cereals, among which highland barley was at a dominating dietary level. As is known, cereals are abundant in carbohydrate and energy rather than in protein and fat (e.g., highland barley in average: 62 g/100 g in carbohydrate and 298 kcal/100 g in energy, but 10.2 g/100 g in protein and 1.2 g/100 g in fat; rice in average: 76 g/100 g in carbohydrate and 352 kcal/100 g in energy, but 12 g/100 g in protein and 0.8 g/100 g in fat) (INFS, C. C., 2017), which would thus lead to a relatively more carbohydrate and less protein intake profiles in their daily diet; and (b) lactation stages. According to the findings of dietary frequency survey, the diet options were varying between either lactation stages. Explicitly, lactating women in general were prone to a monotonous diet pattern in colostrum stage, merely including Zanba, buttered tea, and red meat and bone soup, which could be reflected in the shortage of daily energy, protein, and fat intakes. The diet profiles favored relatively more diversified combination in transitional milk stage, with more occurrence of other cereals, eggs, leafy vegetables, and fruits, which significantly (*p* < .05) enhanced the intake values of energy, fat, and carbohydrate. In mature milk stage, the selection of food species was further more various and individualized in each food category, while such variability was confined to the substitution within the same food category, which did not present obvious differences across different food categories. Moreover, the intake values of energy and fat in mature milk stage were significantly lower than the intakes in transitional milk stage, which might be due to the alteration of maternal nutritional needs since the extended duration of lactation period.

**TABLE 7:** Daily values of macronutrient and energy intakes from maternal diets and their correlation analysis with human milk during lactation

Parameter	Colostrum stage			Transitional milk stage			Mature milk stage			Dietary recommendations
	Mean	*r* ^a^	*p* value^a^	Mean	*r*	*p* value	Mean	*r*	*p* value	
Energy (kcal/d)	1,708.20c^b^	.157	.292	3,248.18a	.032	.847	2,969.03b	.024	.882	2,300−2,900^c^
Protein (g/d)	64.87	−.319	.029	67.57	.417	.008	70.57	.249	.113	80^d^
Fat (g/d)	64.08c	.095	.527	85.33a	−.015	.927	74.79b	−.101	.525	20–30 of % Energy^e^
Carbohydrate (g/d)	203.67b	−.051	.734	233.59a	.069	.678	237.63a	−.128	.417	160^f^

^a^ *r* = correlation coefficient of certain parameter between daily maternal diet and human milk; *p* value = significance level of certain parameter between daily maternal diet and human milk.

^b^ Different lowercase letters after the mean values of a certain parameter in a row represent statistical differences among the three lactation stages, *p* < .05.

^c^ Estimated energy requirement (EER) for lactating mothers, issued by National Health Commission of PR China (WS/T 578.1—2017).

^d^ Recommended nutrient intake (RNI) of protein for lactating mothers, issued by National Health Commission of PR China (WS/T 578.1—2017).

^e^ Acceptable macronutrient distribution range (AMDR) of fat for lactating mothers, issued by National Health Commission of PR China (WS/T 578.1—2017).

^f^ Recommended nutrient intake (RNI) of carbohydrate for lactating mothers, issued by National Health Commission of PR China (WS/T 578.1—2017).

Bivariate correlation analysis was processed upon a homogeneous comparison of macronutrients between daily maternal diet intakes and the corresponding human milk samples by SPSS. Interpretively, there are mainly two constituent parts of carbohydrates in human milk, namely lactose and oligosaccharides. Thereinto, lactose consists the principal proportion (generally over 90%) in human milk to ensure appropriate nutrition and development needs for infants, while oligosaccharides in human milk, primarily as prebiotics, exert different effects in the infant gut (Gridneva et al., 2019). Therefore, it is admissible to correlate lactose in human milk with carbohydrates in maternal diets upon the aspect of macronutrients.

As displayed in Table 7, only the item of protein displayed varying significant degrees of correlation through the three lactation stages, whereas other macronutrients in human milk were uncorrelated with the relevant macronutrients in daily intakes. Such results were not quite parallel with some previous relevant reports (Aumeistere et al., 2019; da Cunha et al., 2005). In this research, the intake of protein was negatively weak‐correlated between human milk and maternal diet in the colostrum stage, then positively medium‐correlated in the transitional milk stage, and finally uncorrelated in the mature milk stage. Meanwhile, protein intakes from maternal daily diet were lower than the values of Chinese RNIs through all the three lactation stages. However, the protein contents in human milk samples did not possess obvious inadequacy accordingly in the corresponding lactation stages (Table 3), which was consistent with some relevant publications (Shi et al., 2011; Zhu et al., 2017). Such consequence might reveal that protein shortage in daily maternal diet was still a restrictive factor for the lactating women, especially in the colostrum and transitional milk stages. Benignantly, the nutritional metabolism involving multiple constituents in maternal digestion tract and mammary gland would be conducive to the maintenance of protein content in human milk. Meanwhile, the overcapacity contents of carbohydrate in maternal daily diets did not induce the excessive proportions of carbohydrate in their human milk, which indicates that the internal metabolism might be more influential than the external maternal dietary profiles to the ultimate composition of human milk.

### 3.8 Correlation analysis of key functional micronutrients between maternal diet profiles and human milk during different lactation stages

**FIGURE 2:**
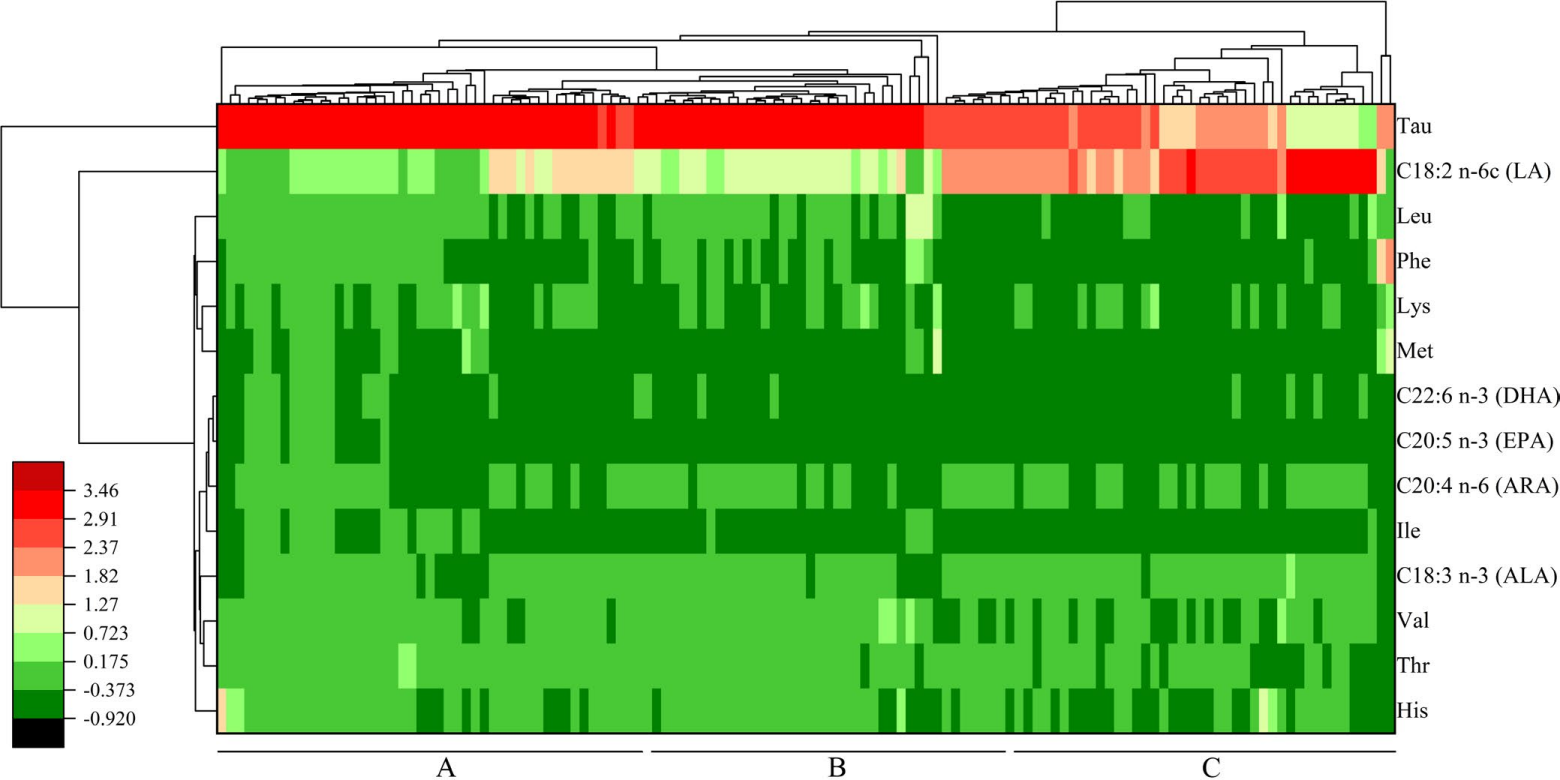
Cluster heatmap analysis of free essential amino acids (*His*, *Leu*, *Lys*, *Phe*, *Val*, *Thr*, *Met*, *Ile* and *Tau*) and main functional unsaturated fatty acids in human milk. LA, linoleic acid, ALA, α‐linolenic acid, ARA, arachidonic acid, EPA, eicosapentaenoic acid, DHA, docosahexaenoic acid. (Group A, Group B, and Group C were divided according to the distributive orders and amounts of human milk samples from different lactation stages). Group A was enriched in colostrum samples (*n*_colostrum_: *n*_transitional milk_: *n*_mature milk_ = 25:11:11, 25 of 47), Group C was enriched in mature milk samples (7:14:21, 21 of 42), while Group B contained quite balanced milk samples from any lactation stage (16:15:10)

Cluster heatmap analysis was applied to show the differences and similarities of the key functional micronutrients in this study among human milk samples from three lactation stages, including free essential amino acids (*His*, *Leu*, *Lys*, *Phe*, *Val*, *Thr*, *Met*, *Ile*, and *Tau*) and main functional unsaturated fatty acids (LA, ALA, ARA, EPA, and DHA), as is shown in Figure 2. The content variations of the key functional micronutrients clustered into three categories. Cluster 1 was consisted of *His*, *Leu*, *Lys*, *Phe*, *Val*, *Thr*, *Met*, *Ile*, and ALA, ARA, EPA, and DHA, the contents of which generally decreased nonmonotonically according to orders of human milk samples; Cluster 2 was consisted of LA, the content of which increased nonmonotonically to a relatively high level accordingly; and Cluster 3 was consisted of *Tau*, the content of which decreased accordingly from a relatively high level. The cluster findings among the human milk samples indicated that the content variations of the involved micronutrients were not distinctly classified according to different lactation stages. However, some potential relevance could be implied when we divided the human milk samples into three groups (as is Group A, Group B, and Group C), according to the distributive orders and amounts of human milk samples from different lactation stages. Group A was enriched in colostrum samples (n_colostrum_: *n*_transitional milk_: *n*_mature milk_ = 25:11:11, 25 of 47), Group C was enriched in mature milk samples (7:14:21, 21 of 42), while Group B contained quite balanced milk samples from any lactation stage (16:15:10). As mentioned above, the maternal diet profiles of lactating mothers were diversified upon different lactation stages, especially between colostrum stage and the other two lactation stages (Figure 1). Consequently, relationship in various degrees between key micronutrients in human milk and maternal diet pattern could be indicated through different lactation stages, which was more obvious between colostrum stage and the other two lactation stages. Additionally, such observations among the involved micronutrients resembled the correlation results of macronutrients with human milk samples as mentioned above.

Previous publications have considered that micronutrients, such as functional unsaturated fatty acids and free essential amino acids, might be more dependent on the daily dietary conditions during lactation (Aumeistere et al., 2018, 2019; Garcia‐Rodenas et al., 2016; Kim et al., 2017). Generally, the food categories of seafoods, eggs, and nuts are important sources for functional unsaturated fatty acids, while such kinds of food were not frequently enough prepared in the participants' daily diets. In consequence, the contents of main functional fatty acids in the human milk samples of this research, including PUFA, *n*−3 fatty acids, and *n*−6 fatty acids, were mostly insufficient than the published data from Chinese urban areas and other similar regions (Cruz‐Hernandez et al., 2013; Jiang et al., 2016; Thakkar et al., 2019). Meanwhile, the supply of FAAs in human milk, especially essential FAAs, which likewise rely on the quality of maternal daily diets (Zhang et al., 2013), was also deficient among the participants in this research than in other more developed areas (Garcia‐Rodenas et al., 2016; Liang et al., 2018).

## 4 LIMITATIONS

This study has its limitations. First, although the effect of maternal diet pattern on human milk composition of macronutrients and micronutrients has been discussed in various studies, the impact of traditional maternal diet patterns on human milk composition at Tibetan high‐altitude areas was poorly documented in recent publications, especially aiming at rural farmer groups. Therefore, few previous relevant reports are available for explicit analysis and clarification. Secondly, the participants in this study were scattered living across the rural areas of Shigatse with limited means of transportation, which brought more difficulty to collect samples and survey files. Consequently, the recruitment and follow‐up survey of fifty effective participants were a realistic attempt to investigate the traditional Tibetan maternal diet pattern and its impact on the human milk samples during different lactation stages, while it would be more considerable if a wider range of cohort research with larger sample capacity was available in future.

## 5 CONCLUSIONS

Traditional maternal diet pattern during the first month postpartum in rural areas of Shigatse, Tibet, was depicted in this research, which was monotonously abundant in Zanba, buttered tea, red meat, and fatty soup, yet inadequate of white meat, eggs, leafy vegetables, and fruits. Accordingly, the nutritional values of maternal diet intakes were high in contents of carbohydrate and deficient in contents of protein when compared to the values of Chinese RNIs. Maternal diet profiles presented distinguishable characteristics in various degrees though different lactation stages, which influenced the composition of human milk. Except for the weak‐to‐medium correlation of protein contents, the composition of maternal diet insignificantly affects other macronutrient levels of human milk during the lactation period. Conversely, micronutrient components, including functional unsaturated fatty acids and free essential amino acids, were impacted by maternal diet conditions, which were in insufficient levels than more developed regions in Chinese urban areas and other similar regions. Thus, more efforts should be required to support the enhancement of local maternal nutritional level and the delicacy cultivation of maternal and infant health care.

## CONFLICT OF INTEREST

The authors declare that they have no known competing financial interests or personal relationships that could have appeared to influence the work reported in this paper.
